# Examining the influence of country-level and health system factors on nursing and physician personnel production

**DOI:** 10.1186/s12960-016-0145-4

**Published:** 2016-08-15

**Authors:** Allison Squires, S. Jennifer Uyei, Hiram Beltrán-Sánchez, Simon A. Jones

**Affiliations:** 1 Rory Meyers College of Nursing, New York University, 433 First Avenue, New York, NY 10010 United States of America; 2 Research on Medical Education Outcomes (ROMEO) Division, School of Medicine, New York University, 433 First Avenue, New York, NY 10010 United States of America; 3 Division of Comparative Effectiveness and Decision Science, New York University School of Medicine, 227 East 30th Street, New York, NY 10016 United States of America; 4 Department of Community Health Sciences, California Center for Population Research, University of California, Los Angeles, 650 Charles E. Young Drive South, Room 41-257 CHS, Los Angeles, CA 53706-1393 United States of America; 5 Population Health, New York University School of Medicine, 227 East 30th Street, New York, NY 10016 United States of America

**Keywords:** Human resources for health, Socioeconomic development, Policy, Global health, Complexity theory, Nurse-to-population ratio, Physician-to-population ratio

## Abstract

**Background:**

A key component to achieving good patient outcomes is having the right type and number of healthcare professionals with the right resources. Lack of investment in infrastructure required for producing and retaining adequate numbers of health professionals is one reason, and contextual factors related to socioeconomic development may further explain the trend. Therefore, this study sought to explore the relationships between country-level contextual factors and healthcare human resource production (defined as worker-to-population ratio) across 184 countries.

**Methods:**

This exploratory observational study is grounded in complexity theory as a guiding framework. Variables were selected through a process that attempted to choose macro-level indicators identified by the interdisciplinary literature as known or likely to affect the number of healthcare workers in a country. The combination of these variables attempts to account for the gender- and class-sensitive identities of physicians and nurses. The analysis consisted of 1 year of publicly available data, using the most recently available year for each country where multiple regressions assessed how context may influence health worker production. Missing data were imputed using the ICE technique in STATA and the analyses rerun in *R* as an additional validity and rigor check.

**Results:**

The models explained 63 % of the nurse/midwife-to-population ratio (pseudo *R*^2^ = 0.627, *p* = 0.0000) and 73 % of the physician-to-population ratio (pseudo *R*^2^ = 0.729, *p* = 0.0000). Average years of school in a country’s population, emigration rates, beds-per-1000 population, and low-income country statuses were consistently statistically significant predictors of production, with percentage of public and private sector financing of healthcare showing mixed effects.

**Conclusions:**

Our study demonstrates that the strength of political, social, and economic institutions does impact human resources for health production and lays a foundation for studying how macro-level contextual factors influence physician and nurse workforce supply. In particular, the results suggest that public and private investments in the education sector would provide the greatest rate of return to countries. The study offers a foundation from which longitudinal analyses can be conducted and identifies additional data that may help enhance the robustness of the models.

**Electronic supplementary material:**

The online version of this article (doi:10.1186/s12960-016-0145-4) contains supplementary material, which is available to authorized users.

## Background

A key component to achieving good patient outcomes is having the right number and type of healthcare professionals with the right resources. Research increasingly demonstrates where and how the healthcare workforce impacts patient outcomes, quality of care, and health inequalities in both primary and acute care settings [1–12]. Political and social determinants of health also contribute to patient outcomes to varying degrees [13].

The production and retention of human resources for health (HRH) at levels that can sustainably achieve good health outcomes seems like it should be the simple case of having enough schools, teachers, and resources to prepare the future healthcare worker to address the healthcare needs of a country’s population, followed by appropriate management and governance that creates supportive practice environments. If it were that simple, Latin America and former Soviet Union countries, for example, would not overproduce physicians and under produce nurses [14]. As regional examples of this trend, the country of Georgia has 3.2 nurses and midwives per 1000 population and 4.8 physicians while Colombia has 0.6 nurses per 1000 population and 1.5 physicians per 1000 population. Navarro et al. [15] produced one of the few studies examining this dynamic, but focused their analysis on health outcomes and not health worker production. Countless other examples from around the world suggest that resources and infrastructure are only a small part of the healthcare human resource production equation.

Therefore, this study seeks to explore if country-level contextual factors have an impact on HRH production and, if so, to what extent. We define country-level contextual factors as those broader institutional structures that affect, directly or indirectly, the healthcare system, population health, and health worker supply and demand. We take the health worker-to-population ratio as a measure of production instead of availability because a measure of health worker availability means the worker is trained and employed as health workers [16]. Since we know health workers may maintain a license or credential even if they are not working in the country (due to domestic un- or underemployment or because they are working abroad) and many countries do not have accurate and updated health worker records (e.g., workers who are alive vs. dead), for the purposes of this paper the measure is used as a marker of production since good quality data about health worker availability is not consistently available. Our focus for this paper is limited to physicians and nurses/midwives since they represent the largest composite group of professional healthcare workers around the world and have the most reliable data sources. We hypothesize that the identified indicators are differentially linked with physician and nurse supply and study these groups separately.

To date, only a few studies have looked at the context of HRH production at a macro-level [17–19]. Squires and Beltrán-Sánchez [20, 21], for example, conducted two studies that demonstrated a link between educational variables at the State level and the nurse-to-population ratio in Mexico, with average years of education correlating strongly with the overall ratio (*R* = 0.69, *p* = 0.001) and to the ratio of nurses with specialty training (e.g., operating room, intensive care, obstetrics) (*R* = 0.54, *p* = 0.020). Generally, however, the literature quantifying HRH production is scarce, comes largely from high-income countries in the form of policy statements, and examines the phenomenon mostly through a supply side lens. At the micro-level, research examines the career intentions of students [22–24], the impact on worker production of teacher shortages [25–27], and education system dynamics that affect supply [28–31]. Research about demand for health workers from epidemiological and demographic forecasts take a broader view but do not consider how a profession itself creates “demand” for individuals to attempt to gain entry into it (via the public image, perceived employment stability, salary, etc.) and other factors that may influence their ability to do so. Thus, exploring production issues just at the micro-level ignores the complex contextual picture that is involved with HRH production. It does not consider how economic indicators, political factors, and social inequality may influence the production process. This analysis will explore the influence of those factors on HRH production using physicians and nurses as examples.

## Methods

The design of this observational study draws from multiple disciplines, including the social sciences, health services research, and public health. The theoretical grounding for the study rests in complexity theory combined with a realist perspective. The combined perspectives “understand reality as comprising multiple, nested, open system in which change is generative, context dependent, and time irreversible,” (Westhorp, [32], p. 406), further positing that causation occurs through mechanisms that are context sensitive, and capture how micro-level interactions translate into macro-level outcomes [32, 33]. In the case of healthcare human resource production, this theory provides the structure for understanding how socioeconomic development, political systems, and other national social factors influence HRH production because it is a context-dependent process involving the interactions between multiple systems.

Variable selection drew from a critical review of interdisciplinary literature from healthcare and the social sciences and expert consultation. They represent population characteristics, health system factors, economic variables known to directly affect health systems, a measure of political stability, and variables accounting for social inequality in a society. In particular, to attempt to account for the effects of gender-sensitive social inequality, we use the United Nations Development Program’s (UNDP) Gender Empowerment Measure (GEM). We selected that variable with the idea that professions that were biased toward one gender or another may have their production affected due to the country’s specific dynamic in that area. The combination of these variables attempts to account for the gender- and class-sensitive identities of physicians and nurses. Our variable selection and rationales for choosing them are found in Table 1.

**Table 1:** Variable selection rationale and data sources

Indicator	Link with HRH production and rationale	Data source^a^
Population characteristics		
Schooling	Average years of school of a country’s population. Educational access is a key to producing health workers.	CIA.gov
Migrate	Migration rates of a country (in and out). Migration is known to affect health workforce supply.	CIA.gov
Urbpop	Urban population percentage. Health workers are known to cluster in urban areas.	CIA.gov
Population health status		
Beds-per-1000 people	Reflects health system capacity for worker employment.	WorldBank.org
Public health exp	Healthcare human resources are the largest expenditure of a health system.	WorldBank.org
Private health exp		WorldBank.org
Economic		
Gini	The Gini index is considered the best measure of economic inequality in a country. Economic inequality affects health outcomes because it may affect access to health workers.	WorldBank.org
WB Income Level	The World Bank classifies countries by four income levels: low income, low middle-income, high middle-income, and high income. It is the best way to control for between country variations in economic status.	WorldBank.org
Debtext	External debt affects what a country can spend on healthcare.	CIA.gov
GDPPP	Gross Domestic Purchasing Power Parity is a proxy measure of national income that better accounts for inequality than GDP per capita.	CIA.gov
Political System [35]	The Polity IV project divides political regimes into three categories: democracy, anocracy, and autocracy. This allows for categorical analysis of governance variables and reduces variability.	http://www.systemicpeace.org/polity/polity4.htm
Social inequality		
GEM	The Gender Empowerment Measure is how the United Nations Development Program attempts to measure gender inequality. It is a composite measure of women’s relative economic income, participation in high-paying positions with economic power, and participation in governance.	UNDP.org

^a^The most recently available years of data were used in the analyses

We use nurse/midwife-to-population ratio (NMPR) and the physician-to-population ratio (PPR) as indicators of HRH production. Combining nurses and midwives into a single aggregated indicator is standard practice for data reporting by the World Health Organization (WHO) because in some countries, nurses are dually trained as midwives or midwives may not have formal training and are categorized as community health workers [34]; therefore, we opted for the aggregated indicator because we felt it was a better representative.

**Fig. 1:**
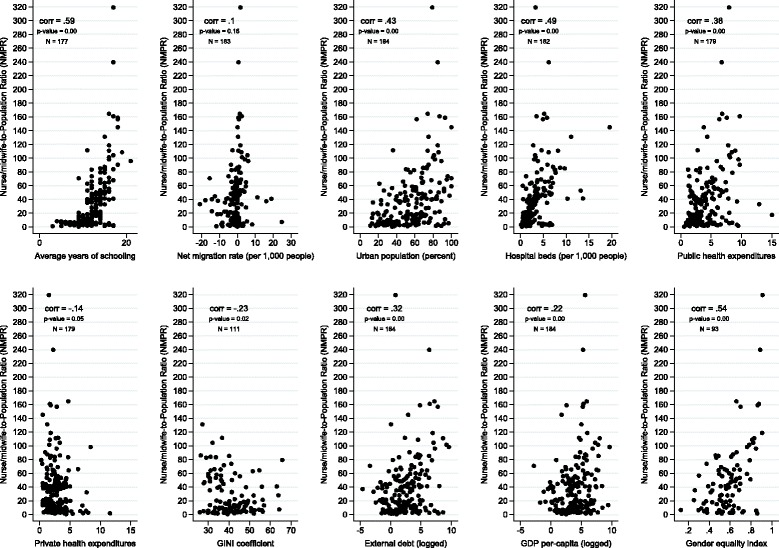
Associations between the Nurse-Midwife-to-Population Ratio and Selected Development Indicators

For each indicator, we then conducted a series of negative binomial regression models at the country level while controlling for factors shown in Table 1 and using the country’s population size as an offset. The latter step allows us to control for differences in population size across countries. We began the analysis by dividing the variables into five groups as shown in Table 1: population characteristics, health system, political system, economic, and social inequality. Logs were generated for the economic variables “external debt” and “Gross Domestic Purchasing Power Parity (GDPPP)” as a way to reduce variability, a standard practice in economic analyses. For the polity variable, we used the three categories recommended by the Polity IV project for analysis: democracy, anocracy, and autocracy [35]. Figure 1 from the Polity IV project illustrates the 2011 categorizations of global states, which have remained stable since that year.

We began with univariate and bivariate analyses and then proceeded to the multivariate models. Multicollinearity checks based on the variance inflation factor suggested no collinearity issues since all measures were less than ten. The first model included population and health system characteristics, as they represent the variables closest to the actual healthcare worker. We then tested the impact of political and economic variables on the model in the second iteration. The final model included a social inequality indicator variable, the gender empowerment measure (GEM), which is commonly used in United Nations analyses of gender.

The majority of our independent variables had less than 4 % missing data, but three had substantial data missing (polity 14 %, Gini 40 %, and GEM 49 %). All variables were imputed using chained equation (ICE), a technique available in STATA software, and models were run with and without imputation to check the degree to which the results were influenced by imputation. A pseudo *R*^2^ result was produced to explain the variation in a model because in the case of this analysis, no direct ways of estimating *R*^2^ exist. Overall, results were robust to the imputation approach with consistent coefficients in magnitude and direction with and without imputation. All analyses were conducted in STATA, then rerun in *R* as a validity check. Results from additional checks related to multicollinearity and the imputation approach can be found in the "Additional File 1" online.

## Results

**Fig. 2:**
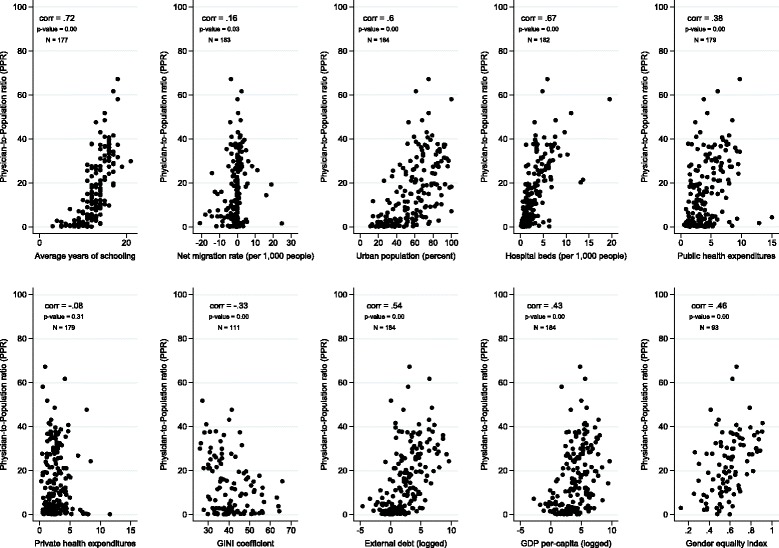
Associations between the Physician-to-Population Ratio and Selected Development Indicators

Figures 1 and 2 illustrate the bivariate relationships between the independent and dependent variables for nurses and physicians, respectively. All variables were significantly correlated with the NMPR, except for the net migration rate (Fig. 1). Private sector health expenditure and the Gini coefficient were negatively correlated with the NMPR at *R* = −0.15, *p* = 0.04, and *R* = −0.26, *p* = 0.03, respectively. For the PPR, net migration rate was positively and significantly linked but only slightly correlated (*R* = 0.16, *p* = 0.03) while private sector health expenditure was not correlated (*R* = −0.07, *p* = 0.29) (Fig. 2). Average years of schooling (*R* = 0.60, *p* = 0.00), GEM (*R* = 0.53, *p* = 0.00), and beds-per-1000 population (*R* = 0.49, *p* = 0.00) were the top three variables positively correlating with the NMPR. Similarly, the PPR also had average years of schooling (*R* = 0.71, *p* = 0.00) and beds-per-1000 population (*R* = 0.65, *p* = 0.00) in the top three, but urban population percentage (*R* = 0.60, *p* = 0.00) beat the GEM’s correlation (*R* = 0.46, *p* = 0.00) by 14 points. Gini coefficients had identical negative and significant correlations with the NMPR (*R* = −0.26, *p* = 0.03) and PPR (*R* = −0.26, *p* = 0.03). Further, external debt rates, while both significant, were twice as correlated to the PPR (*R* = 0.50, *p* = 0.00) as the NMPR (*R* = 0.25, *p* = 0.00).

Table 2 illustrates the results from the negative binomial regressions for nurses and physicians. The final model explained 63 % of the NMPR (*R*^2^ = 0.627, *p* = 0.0000) and 73 % of the PPR (*R*^2^ = 0.729, *p* = 0.000) based on the maximum likelihood pseudo *R*^2^ [36]. Notably, for every additional year spent in school, a country’s NMPR increases by 18 % (*p* = 0.001) and PPR increases by 19 % (*p* = 0.001). These results were consistently significant across the models. The beds-per-1000 population were also significant predictors of the total nurse/midwife (11 % increase, *p* = 0.001) and physician (14.5 % increase, *p* = 0.001) numbers and consistent across the models. Emigrant population rate was also significant for both types of providers, indicating that relative to countries with no migration, those with negative migration flows have significantly higher nursing (*p* = 0.05) and physician (*p* = 0.001) populations. Unsurprisingly, status as a low-income country relative to high income reduces nurse and physician supply by 30 % and was significant for both (*p* = 0.001).

**Table 2:** Coefficient estimates expressed as incidence rate ratios (IRR) from a negative binomial model associating nurse/midwife-to-population ratio (NMPR) and physician-to-population ratio (PPR) with macro-level variables at the country level

Variable	NMPR			PPR		
	Model 1	Model 2^a^	Model 3^a^	Model 1	Model 2^a^	Model 3^a^
Population characteristics						
Schooling (years)	1.216***	1.183***	1.185***	1.221***	1.192***	1.189***
Migrate (ref = zero migration)						
Emigrant	1.460*	1.441*	1.458*	2.121***	1.848***	1.757***
Immigrant	1.434	1.341	1.321	1.641**	1.449	1.456
Urban population (%)	1.003	0.999	0.999	1.019***	1.010**	1.010**
Health system characteristics						
Beds-per-1000 people	1.104***	1.108***	1.110***	1.167***	1.151***	1.145***
Public health exp	1.009	1.011	1.006	0.941	0.934*	0.953
Private health exp	0.954	0.993	0.992	1.03	1.054	1.061
Political system (ref = democracy)						
Autocracy		1.045	1.042		0.943	0.94
Anocracy		1.407	1.421*		1.17	1.137
Economy						
Gini		1	1		0.995	0.996
WB income (ref = high)						
Middle		0.699	0.709		0.902	0.837
Low		0.322***	0.333***		0.339***	0.303***
External debt		0.958	0.958		1.009	1.014
GDPPPP		1.003	1		1.052	1.058
Social inequality						
GEM			1.243			0.461
Sample size	173	184	184	173	184	184
Maximum likelihood *R* ^2^	0.566	0.625	0.627	0.651	0.721	0.729

*WB* World Bank, *GEM* gender empowerment measure, *GDPPPP* GDP per person

***p < 0.001; **p < 0.01; *p < 0.05

^a^Missing data in income was imputed using the ICE method

In addition, one variable was only significant for physician supply. Urban population percentage was a significant production predictor across the models for physicians (*p* = 0.01), but not for nurses. Also notable, a country categorized as an “anocracy,” relative to democracy, increases its nurse supply numbers by 42 % (*p* = 0.050) but has no effect on physician supply.

With the exception of low-income country status, none of the other economic variables selected for this study were significant predictors of nurse or physician supply in the fully adjusted models; however, the models suggest some important trends to study further. A one-point increase in GDPPP translates into 5 % more physicians but adds nothing to nursing supply. External debt has an opposite effect for nurses and physicians suggesting a negative effect on the nursing supply, reducing nurses by 4 %, but a positive one on physician supply adding 1.4 % more. Economic inequality, as measured by the Gini coefficient, appears to have a minute effect on decreasing physician supply but no effect on nursing numbers.

## Discussion

The strength of the models and their ability to explain 63 to 73 % of the relationship between context and production of the two largest health workforce cadres offers striking new insight into the complex set of dynamics involving health workforce production. For some countries, a grim picture may be present in these results, but the findings do provide some clearer direction for policy initiatives. They also suggest that targeted investments aimed at meeting the sustainable development goals (SDGs) may translate both directly (e.g., quality education, gender equality, good health, and well-being) and indirectly (e.g., no poverty, zero hunger, clean water, and sanitation) into improved health workforce production. More skilled and competent health workers are essential for meeting many of the SDGs.

In consideration of the models, notably comparing pseudo *R*^2^ for models with different outcomes should be carried out with caution. To partially mitigate this risk, two other methods for estimating pseudo *R*^2^ were used [37, 38] and a comparable difference was still observed. The 10 % difference in variance explained between physicians and nurses in the model, we believe, cannot be explained by existing data because of the amount of cross-national inconsistencies in information systems about healthcare human resources [39, 40]. Per Riley et al. [40], even if new data were collected, it may not be consistent, of good quality, nor vetted by internationally accountable authority.

In light of the aforementioned data availability and quality issues, an additional variable that may help align the NMPR model to results that are equivalent to the PPR model, along with enhancing its robustness, is the number of nursing and medical schools in each country. Publicly available data about the number of educational institutions would also help to further refine the explanation of the strong correlation and significance of the “average years spent in school” of a country’s population and the health workforce ratios. Furthermore, Owen and You [41] found that the strength of institutions, like education, helps reduce gender inequality. Equitable access to education contributes to HRH production, primarily by ensuring women’s ability to obtain it. Even though the University of Copenhagen maintains a list of the world’s medical schools [42], the same data are not available for nursing nor other healthcare professions; neither WHO, the International Council of Nurses, nor comparable organizations have this information publicly available.

An additional education-sensitive, model-enhancing factor specific to nursing personnel would be accounting for the educational variation within the profession. Since nursing education around the world can range from post-secondary through the doctoral level, more precise data about degree composition within the nursing profession at the country level would strengthen the precision of the model, not to mention patient outcome analyses and workforce planning. It would be very helpful for quantifying faculty shortages which will affect production. Data of this type could also provide strategic direction for the best types of degree programs to open in a country in order to facilitate production that can efficiently meet population health needs through universal health coverage and generate optimal outcomes. This model could help provide the evidence for those types of policy changes.

Thus, for enhancing HRH data quality across the professions (and subsequently the quality of future analyses), it would be ideal to capture the total number of schools in a country, faculty and their educational preparation, categories of education (e.g., vocational/practical, bachelors, masters), entry-level education requirements, graduation rates, and employment rate post-graduation. The presence of a licensure exam or similar credentialing process would also be useful. These data would enhance not only labor market analyses but sociological ones and health outcomes analyses.

Despite the challenges of the data and considering other potential explanations for the findings, the dynamics of professional ecology may help explain the differences in the results between physicians and nurses. Medicine has created itself into a socially elite profession around the world. The positions of power and authority physicians hold in many countries ensure that elites and social class mobile individuals will study medicine there, even if they do not stay. Nursing, however, presents an entirely different story. Squires et al. [43] posit that a negative public image of nursing—resulting from a combination of low salaries, poor work environments, female gender association, and, in some places, a lack of career mobility that deters the twenty-first century applicant—creates a cyclic effect that hampers the production of nurses. Social mores around acceptable careers for women and their work schedules (i.e., night shift and its perceived safety for female workers) may further contribute to production issues for nursing personnel. Cross-national data reflecting public opinion of the nursing profession would help to determine the validity of that hypothesis and may help to further explain the ten-point gap in the results of the models between the two professions.

Other aspects of the models allude to the challenges of health system management. Regardless of location, health workers need places to work and bed availability for the population appears to be a proxy indicator of employment opportunity for both physicians and nurses. These results require further study and refinement, however, since nurses may staff primary healthcare systems at greater rates in low-income countries. Furthermore, the total number of listed beds a country reports may not all be in service due to health system financing issues. Future studies that better account for the number of beds-in-use and public primary care clinics in a country may help refine the result.

The significance of the economic and inequality variables in the model suggests that systematic national policies aimed at reducing social, gender, and economic inequality could positively affect health workforce production. It may also explain the finding about the relationship between the NMPR and migration rates since men are more likely to migrate than women [44], women are more likely to follow their migrating husbands than initiate it themselves [45], and low- and middle-income source countries for international nurse migration are dominated by a small number of countries [46]. This may prove especially important for nursing workforce production because of its female gender dominance. Previously, most studies of gender issues in the health professions, besides the aforementioned educational access ones, centered largely on pay disparities between men and women in each occupational group and largely focus on high-income countries [47–51]. Our results indicate that nurse/midwife production may be more sensitive to broader gender inequality issues than physicians. In some ways, this may seem like a “common sense” finding, but research had not previously quantified it.

Finally, when conducting the analyses, we found complexity theory a useful framework for considering variable relationships and selection. Since we aimed to avoid a “kitchen sink” approach to conducting the multivariable regressions, the theory provided direction for how to prioritize variables given our collective, interdisciplinary backgrounds and have the models reflect the complex, interconnected systems that are health services delivery and HRH production.

From a policy perspective, to the best of our knowledge, ours is among the first papers to quantify the significance of how national education policies may impact a country’s healthcare worker production. We have known for many decades that education matters for population health, and now, the significance of the relationship is quantified. Our results illustrate the importance of concurrent policy efforts between education and healthcare sectors for stable HRH production and support statements by Frenk et al. [52]. Results from this paper can also be used to help meet international midwifery production goals, as described in the landmark Lancet series published in June of 2014 [53]. The results suggest that policymakers that increase investments in primary and secondary education are likely to see payoffs in increased health worker production.

This analysis is also among the first to highlight how different political regimes and governance issues influence health workforce production. Our results support findings from the social sciences that have shown positive effects of egalitarian regimes on broadly reducing inequality, both economic and gender-based [54]. Finally, the results further help to delineate the nature of political actors who shape healthcare policy, as recommended by van Olmen et al. [55], along with supporting Varghese and Kutty’s [56] work on improving governability in public health systems. Findings may also inform the movement toward global health governance by illustrating the impact of States’ political systems and governance practices on health workforce production.

In terms of study limitations, known data quality issues in health and development datasets may have affected our results overall due to inconsistencies in reporting across countries and data coordination failures known to affect cross-national datasets [39, 40, 57]. The choice to conceptualize the study on production versus availability was due to the data issues and has its own limitations. For nursing supply data in particular, how countries define nurses and midwives can create fluid interpretations of who qualifies as nursing personnel, and calculations of nursing supply data are not the same across countries [39, 58]. The beds-per-1000 population data also do not account for beds not in service in the country, which may affect employment opportunities for both physicians and nurses and reflect the financial health of the system. Differentiation between hospital-based and primary care-based employment was also not possible to obtain through publicly available sources; those data would also add tremendously to the precision of the analysis in future studies. Polity, Gini, and GEM data had substantial data missing and so those variables required more imputation than others in the study, which may have affected the results. Nonetheless, coefficient estimates for the main factors associated with nurses and physicians (e.g., education, migration, and beds-per-1000 population) are of similar magnitude and direction before and after the imputation, suggesting that our substantive results are not driven by the imputation method.

## Conclusions

In conclusion, this study offers a foundation for studying how macro-level contextual factors influence physician and nurse workforce supply. Additional analyses that use this model and add the health worker ratios as independent variables with patient outcomes as the dependent variables will also make for useful contributions to the literature. Future research should also include replicating the study with pharmacy and dental personnel which would be a logical next step to check the stability of the model across professions. When more data becomes publicly available, replication of these cross-country comparative analyses will allow researchers to further refine the methodologies and more precisely account for the impact of potentially confounding factors related to production. Since HRH data quality is improving because its value for policymakers has increased in the last decade, panel data analyses will also become feasible. Event analyses, for example, that can account for major economic and political changes will also allow researchers to study the impact of those events on HRH production over time. Future studies may also want to test other variables in the categories we identified to see if these enhance the model’s precision. Hence, for strategic workforce planning initiatives and global health governance, this study makes a useful contribution to the policy dialogue.

## Abbreviations

GEM, Gender Empowerment Measure; HRH, human resources for health; ICE, imputation using chained equations; NMPR, nurse/midwife-to-population ratio; PPR, physician-to-population ratio; UNDP, United Nations Development Program; WHO, World Health Organization

## Additional file

Additional file 1: Supplementary results related to multicollinearity and data imputation. (DOC 25 kb)
