# Supplementary material for: Examining the influence of country-level and health system factors on nursing and physician personnel production
Source: Hum Resour Health. 2016 Aug 15;14:48. doi: 10.1186/s12960-016-0145-4 (PMC4983794; doi:10.1186/s12960-016-0145-4)
Supplement: Supplementary file 1 [file 12960_2016_145_MOESM1_ESM.doc]

Variable	NMPR						PPR					
	Model 1		Model 2[a]		Model 3[b]		Model 1		Model 2[a]		Model 3[b]	
	Coeff	95% CI	Coeff	95% CI	Coeff	95% CI	Coeff	95% CI	Coeff	95% CI	Coeff	95% CI
Population characteristics												
Schooling (yrs)	1.216***	(1.146,1.291)	1.183***	(1.118,1.252)	1.185***	(1.119,1.255)	1.221***	(1.148,1.299)	1.192***	(1.126,1.262)	1.189***	(1.115,1.242)
Migrate (ref=zero migr)												
Emigrant	1.460*	(1.080,1.973)	1.441*	(1.080,1.923)	1.458*	(1.089,1.952)	2.121***	(1.569,2.867)	1.848***	(1.401,2.436)	1.757***	(1.262,2.227)
Immigrant	1.434	(0.966,2.127)	1.341	(0.904,1.989)	1.321	(0.890,1.962)	1.641**	(1.127,2.388)	1.449	(0.997,2.106)	1.456	(0.917,1.943)
Urban population (%)	1.003	(0.996,1.009)	0.999	(0.992,1.006)	0.999	(0.992,1.006)	1.019***	(1.012,1.026)	1.010**	(1.003,1.017)	1.010**	(1.001,1.015)
Health system												
Beds per 1,000 people	1.104***	(1.043,1.170)	1.108***	(1.052,1.168)	1.110***	(1.053,1.170)	1.167***	(1.097,1.242)	1.151***	(1.090,1.216)	1.145***	(1.084,1.206)
Public health exp	1.009	(0.949,1.073)	1.011	(0.952,1.073)	1.006	(0.945,1.071)	0.941	(0.884,1.002)	0.934*	(0.882,0.990)	0.953	(0.935,1.057)
Private health exp	0.954	(0.888,1.025)	0.993	(0.925,1.066)	0.992	(0.923,1.065)	1.03	(0.953,1.114)	1.054	(0.979,1.134)	1.061	(0.991,1.147)
Political system (ref=democracy)												
Autocracy			1.045	(0.783,1.396)	1.042	(0.780,1.393)			0.943	(0.710,1.251)	0.94	(0.699,1.230)
Anocracy			1.407	(0.996,1.987)	1.421*	(1.003,2.014)			1.17	(0.840,1.630)	1.137	(0.830,1.593)
Economy												
GINI			1	(0.993,1.007)	1	(0.993,1.007)			0.995	(0.988,1.003)	0.996	(0.998,1.004)
WB-income (ref=high)												
Middle			0.699	(0.473,1.031)	0.709	(0.478,1.052)			0.902	(0.633,1.284)	0.837	(0.541,1.101)
Low			0.322***	(0.188,0.551)	0.333***	(0.192,0.576)			0.339***	(0.210,0.547)	0.303***	(0.163,0.438)
External debt			0.958	(0.876,1.046)	0.958	(0.876,1.047)			1.009	(0.930,1.096)	1.014	(0.949,1.115)
gdpppp			1.003	(0.913,1.100)	1	(0.910,1.099)			1.052	(0.965,1.148)	1.058	(0.983,1.165)
Social inequality												
GEM					1.243	(0.554,2.788)					0.461	(0.147,0.667)
Sample size	173		184		184		173		184		184	
BIC	3762.0		3979.0		3983.6		3402.6		3580.7		3581.2	
AIC	3733.6		3927.5		3929.0		3374.2		3529.3		3526.6	
McFadden's Adj R2	0.033		0.035		0.035		0.046		0.053		0.054	
Maximum Likelihood R2	0.566		0.625		0.627		0.651		0.721		0.729	
Cragg & Uhler's R2	0.566		0.625		0.627		0.651		0.721		0.731	
